# Supplementary material for: Variation in (Hyper)Polarizability of H2 Molecule in Bond Dissociation Processes Under Spatial Confinement
Source: Molecules. 2024 Dec 24;30(1):9. doi: 10.3390/molecules30010009 (PMC11721705; doi:10.3390/molecules30010009)
Supplement: Supplementary file 1 [file molecules-30-00009-s001.zip › molecules-3360654-supplementary.pdf]

Supplementary Materials

Variation in (Hyper)Polarizability of H_2 Molecule in Bond Dissociation Processes Under Spatial Confinement

Table S1. Total energy of the H_2 molecule obtained as a function of the internuclear distance (R). The calculations have been performed at the CCSD/d-aug-cc-pV6Z level of theory. ω defines the strength of the spatial confinement. All values are given in a.u.

R[a.u]	Energy [a.u]			
	ω=0.00	ω=0.10	ω=0.40	ω=0.80
1.0393	-1.13596705	-1.12307476	-0.95947949	-0.57064868
1.1338	-1.15596377	-1.14253173	-0.97343603	-0.57520998
1.1372				-0.57521483
1.2283	-1.16755558	-1.15359203	-0.97918991	-0.57214014
1.2731			-0.97977062	
1.3228	-1.17311392	-1.15862931	-0.97913346	-0.56384772
1.3871		-1.15949113		
1.4012	-1.17436086			
1.4173	-1.17431405	-1.15932086	-0.97495958	-0.55203522
1.5118	-1.17236256	-1.15687516	-0.96789033	-0.53793031
1.6063	-1.16814183	-1.15217644	-0.95882181	-0.52243144
1.7008	-1.16230582	-1.14588037	-0.94842001	-0.50620393
1.7952	-1.15534454	-1.13847860	-0.93718542	-0.48974439
1.8897	-1.14762849	-1.13034322	-0.92549751	-0.47342466
2.0787	-1.13099687	-1.11294221	-0.90185461	-0.44224990
2.2677	-1.11397644	-1.09525336	-0.87909621	-0.41415755
2.4566	-1.09750161	-1.07821945	-0.85816317	-0.38989365

2.6456	-1.08212928	-1.06240250	-0.83957670	-0.36969885
2.8346	-1.06817958	-1.04812348	-0.82357139	-0.35345607
3.0236	-1.05581508	-1.03554058	-0.81017203	-0.34080023
3.2125	-1.04508707	-1.02469506	-0.79924729	-0.33121939
3.4015	-1.03596444	-1.01554070	-0.79055678	-0.32414739
3.5905	-1.02835435	-1.00796601	-0.78379649	-0.31903710
3.7795	-1.02212001	-1.00181425	-0.77864046	-0.31540783
3.9684	-1.01709752	-0.99690269	-0.77477364	-0.31286630
4.1574	-1.01311181	-0.99304037	-0.77191377	-0.31110633
4.3464	-1.00999020	-0.99004272	-0.76982223	-0.30989802
4.5353	-1.00757256	-0.98774164	-0.76830609	-0.30907376
4.7243	-1.00571738	-0.98599115	-0.76721455	-0.30851420
4.9133	-1.00430443	-0.98466912	-0.76643273	-0.30813575
5.1023	-1.00323461	-0.98367631	-0.76587481	-0.30788018
5.2912	-1.00242828	-0.98293395	-0.76547764	-0.30770698
5.4802	-1.00182259	-0.98238064	-0.76519529	-3.07587948
5.6692	-1.00136870	-0.98196916	-0.76499464	-0.30750375
5.8582	-1.00102910	-0.98166358	-0.76485193	-0.30744158
6.0471	-1.00077523	-0.98143681	-0.76475022	-0.30739354
6.2361	-1.00058547	-0.98126853	-0.76467747	-0.30735524
6.4251	-1.00044358	-0.98114357	-0.76462514	-0.30732450
6.6140	-1.00033737	-0.98105068	-0.76458717	-0.30730019

Table S2. Polarizability (α_{xx}) of the H_2 molecule obtained as a function of the internuclear distance (R). The calculations have been performed at the CCSD/d-aug-cc-pV6Z level of theory. ω defines the strength of the spatial confinement. All values are given in a.u.

R[a.u]	polarizability α_{xx} [a.u]			
	ω = 0.00	ω = 0.10	ω = 0.40	ω = 0.80
1.0393	4.289	4.162	3.334	2.583
1.1338	4.784	4.637	3.706	2.877
1.1372				2.888
1.2283	5.318	5.150	4.106	3.193
1.2731			4.306	
1.3228	5.892	5.699	4.535	3.531
1.3871		6.094		
1.4012	6.397			
1.4173	6.504	6.285	4.989	3.887
1.5118	7.153	6.905	5.468	4.257
1.6063	7.837	7.558	5.968	4.638

1.7008	8.553	8.239	6.484	5.023
1.7952	9.296	8.946	7.012	5.405
1.8897	10.062	9.672	7.543	5.775
2.0787	11.636	11.156	8.588	6.444
2.2677	13.211	12.627	9.545	6.959
2.4566	14.708	14.008	10.335	7.262
2.6456	16.041	15.212	10.887	7.325
2.8346	17.123	16.154	11.154	7.165
3.0236	17.880	16.771	11.129	6.833
3.2125	18.269	17.028	10.846	6.401
3.4015	18.287	16.934	10.371	5.937
3.5905	17.969	16.535	9.782	5.491
3.7795	17.384	15.906	9.154	5.093
3.9684	16.618	15.134	8.544	4.756
4.1574	15.758	14.299	7.988	4.480
4.3464	14.876	13.468	7.503	4.260
4.5353	14.028	12.687	7.095	4.088
4.7243	13.249	11.983	6.758	3.955
4.9133	12.558	11.368	6.483	3.852
5.1023	11.959	10.842	6.268	3.774
5.2912	11.452	10.401	6.095	3.713
5.4802	11.025	10.035	5.958	3.666
5.6692	10.671	9.734	5.851	3.630
5.8582	10.381	9.488	5.767	3.601
6.0471	10.142	9.288	5.700	3.578
6.2361	9.948	9.127	5.648	3.560
6.4251	9.790	8.996	5.607	3.545
6.6140	9.662	8.890	5.574	3.532

Table S3. Second hyperpolarizability (γ_{xxxx}) of the H_2 molecule obtained as a function of the internuclear distance (R). The calculations have been performed at the CCSD/d-aug-cc-pV6Z level of theory. ω defines the strength of the spatial confinement. All values are given in a.u.

R[a.u]	hyperpolarizability γ_{xxxx} [a.u]			
	ω=0.00	ω=0.10	ω=0.40	ω=0.80
1.0393	348.6	300.4	123.7	50.9
1.1338	420.9	359.9	145.4	59.2
1.1372				59.4
1.2283	502.2	427.5	169.7	68.3
1.2731			182.1	
1.3228	599.2	504.1	196.6	79.8
1.3871		561.8		
1.4012	688.9			
1.4173	708.1	593.0	227.9	67.9
1.5118	828.8	685.4	266.0	107.0
1.6063	972.9	791.6	300.2	124.8
1.7008	1135.0	925.7	346.8	143.6
1.7952	1300.4	1065.6	390.1	170.9
1.8897	1507.9	1225.4	459.4	203.1
2.0787	1990.5	1616.7	618.3	292.0
2.2677	2630.6	2104.5	856.8	425.8
2.4566	3452.8	2800.1	1181.3	608.9
2.6456	4548.8	3709.9	1629.8	824.4
2.8346	5978.7	4880.1	2169.3	1033.8
3.0236	7626.7	6305.0	2747.7	1188.1
3.2125	9515.2	7890.8	3269.7	1263.3
3.4015	11495.0	9498.8	3649.6	1275.0
3.5905	13255.0	10858.0	3834.7	1249.5
3.7795	14621.0	11871.0	3829.6	1103.9
3.9684	15583.0	12416.0	3675.6	922.9
4.1574	15761.0	12475.0	3384.4	839.4
4.3464	15575.0	12099.0	3062.5	728.7
4.5353	14876.0	11467.0	2725.1	620.0
4.7243	13942.0	10675.0	2400.8	529.9
4.9133	12835.0	9683.1	2079.0	441.3
5.1023	11668.0	8758.7	1831.7	364.6
5.2912	10509.0	7806.8	1603.9	326.8
5.4802	9435.6	7092.0	1380.3	295.1
5.6692	8438.1	6244.3	1232.6	267.3
5.8582	7573.3	5605.6	1051.3	236.0
6.0471	6744.2	4997.3	978.8	242.4
6.2361	6174.9	4533.2	872.3	199.3
6.4251	5622.2	4098.5	829.2	188.8

6.6140	5137.2	3758.4	755.6	172.5

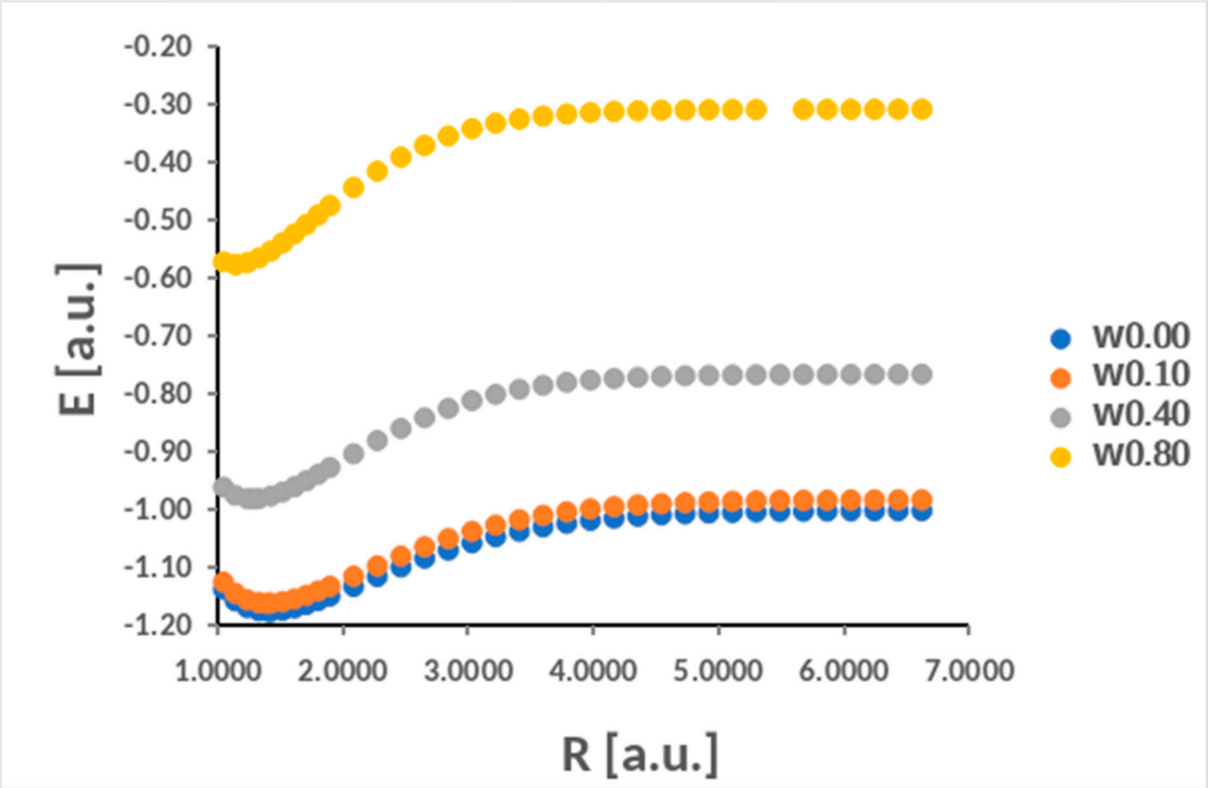

Figure S1. Potential energy curves of the H_2 molecule obtained as a function of the internuclear distance (R). The calculations have been performed at the CCSD/d-aug-cc-pV6Z level of theory. ω defines the strength of the spatial confinement. All values are given in a.u.
